# A computational tool for automatic selection of total knee replacement implant size using X-ray images

**DOI:** 10.3389/fbioe.2022.971096

**Published:** 2022-09-29

**Authors:** Thomas A. Burge, Gareth G. Jones, Christopher M. Jordan, Jonathan R.T. Jeffers, Connor W. Myant

**Affiliations:** ^1^ Dyson School of Design Engineering, Imperial College, London, United Kingdom; ^2^ MSk Lab, Imperial College, London, United Kingdom; ^3^ Imperial College Healthcare, London, United Kingdom; ^4^ Department of Mechanical Engineering, Imperial College, London, United Kingdom

**Keywords:** total knee replacement, medical implants, computer assisted surgery, automated workflows, pre-operative assessment, convolutional neural networks, machine learning

## Abstract

**Purpose:** The aim of this study was to outline a fully automatic tool capable of reliably predicting the most suitable total knee replacement implant sizes for patients, using bi-planar X-ray images. By eliminating the need for manual templating or guiding software tools via the adoption of convolutional neural networks, time and resource requirements for pre-operative assessment and surgery could be reduced, the risk of human error minimized, and patients could see improved outcomes.

**Methods:** The tool utilizes a machine learning-based 2D—3D pipeline to generate accurate predictions of subjects’ distal femur and proximal tibia bones from X-ray images. It then virtually fits different implant models and sizes to the 3D predictions, calculates the implant to bone root-mean-squared error and maximum over/under hang for each, and advises the best option for the patient. The tool was tested on 78, predominantly White subjects (45 female/33 male), using generic femur component and tibia plate designs scaled to sizes obtained for five commercially available products. The predictions were then compared to the ground truth best options, determined using subjects’ MRI data.

**Results:** The tool achieved average femur component size prediction accuracies across the five implant models of 77.95% in terms of global fit (root-mean-squared error), and 71.79% for minimizing over/underhang. These increased to 99.74% and 99.49% with ±1 size permitted. For tibia plates, the average prediction accuracies were 80.51% and 72.82% respectively. These increased to 99.74% and 98.98% for ±1 size. Better prediction accuracies were obtained for implant models with fewer size options, however such models more frequently resulted in a poor fit.

**Conclusion:** A fully automatic tool was developed and found to enable higher prediction accuracies than generally reported for manual templating techniques, as well as similar computational methods.

## 1 Introduction

Prior to total knee replacement (TKR) procedures, surgeons often manually evaluate the size and morphology of patient femur and tibia bones using X-rays taken during preoperative assessment (Tanzer and Makhdom, 2016). This information is used to estimate the most appropriately sized TKR implant components that will be required to achieve both a good global fit, and minimize local areas of over/underhang (OUH), typically from between 5 – 8 sizes (Hitt et al., 2003; Wernecke et al., 2012). Poor implant size selection can result in increased rates of complications, revisions, and patient pain post-surgery (Culler et al., 2017; Schroeder and Martin, 2019; Buller et al., 2018). The presence of regions of OUH ≥3 mm in particular is highlighted as being clinically significant and directly linked with cases of increased soft-tissue irritation, bleeding, osteolysis, laxity in flexion, subsidence, and instability (Schroeder and Martin, 2019; Dai et al., 2014; Shao et al., 2020). Despite the importance of achieving a good fit, the accuracy of manual X-ray assessment and templating, for both femur components and tibia plates, can be poor. Hernández-Vaquero et al. (2019) summarized the reported accuracies from 10 different studies and found mean selection accuracies of 59.2% for femur components and 60.7% for tibia plates. With ±1 size permitted, mean scores of 97.4% and 96.4% were recorded respectively. Consequently, surgeons often opt to implant a different size than estimated during preoperative assessment (Sheth et al., 2017). This could lead to a higher chance of human error, as well as longer procedures.

To attempt to improve the accuracy and reliability of pre-operative size selection, other techniques besides manual X-ray assessment and templating have been explored. Using computerized tomography (CT) imaging was investigated by Vaishya et al. (2018) and Kobayashi et al. (2012). The former reported accuracies of 66% for femur components and 72% for tibia plates, whilst the latter reported an accuracy of just 59% for both. Using demographic factors such as patient height and shoe size as a means of predicting implant size was investigated by Trainor et al. (2018). They reported accuracies of 56/58% for femur components and 56/63% for tibia plates respectively. Sershon et al. (2017) used a combination of demographic variables including height, weight, and sex to build a multivariate linear regression model that achieved ±1 size prediction accuracies of 71–92% and 81–97% for a range of femur component and tibia plate models. Optimum size prediction accuracies were not reported. Limited computational tools capable of predicting TKR implant sizes from X-rays were identified. Zheng et al. (2018) developed an X-ray based tool for pre-operative knee prosthesis planning named “3X.” The authors reported size selection accuracies of 78% for femur components and 70% for tibia plates, based on a study featuring 23 subjects. Massé and Ghate (2021) more recently developed a similar tool named “X-Atlas”, primarily aimed at creating patient-matched cutting guides from X-ray images. The study involved 45 subjects and utilized the Zimmer Biomet “Persona” knee implant. Size selection accuracies of 53.3% for femur components and 57.8% for tibia plates were reported. Both these tools feature “semi-automatic” workflows that require users to manually identify landmarks on the inputted X-ray images and guide “live-wire algorithms” to extract the required bone contours.

In recent years, prediction and classification tools have been developed in other biomedical imaging applications using machine learning techniques to enable automatic workflows. U-Net convolutional neural networks (CNNs) in particular are growing in popularity as highly accurate classification and segmentation models can be trained with a relatively low number of reference images (Ronneberger et al., 2015). Example applications that have adopted this technology include in the diagnosis of COVID-19 (Mohammed et al., 2021) (Narin et al., 2021) and for detecting tuberculosis (Liu et al., 2018). In these studies, CNNs were trained to recognize features and patterns in X-ray images and extract the necessary information for diagnosis, all without requiring any guidance from the user. Furthermore, Cernazanu-Glavan and Holban (2013) demonstrated the benefits of usings CNNs for X-ray-based bone segmentation over alternative methods such as artificial neural networks, principal component analysis and fuzzy clustering. The authors highlighted the superior accuracy of CNNs and that all other methods investigated required some level of human intervention. In the case of computer assisted TKR implant size selection, the tools outlined in previous studies all featured workflows requiring trained users’ feedback (Zheng et al., 2018; Massé and Ghate, 2021). Consequently, the authors do not believe that these “semi-automatic” solutions adequately address the issues preventing widespread adoption of computer assisted sizing tools in TKR. The aim of this study was therefore to develop a proof-of-concept, “fully” automatic alternative, facilitated by employing CNNs in the workflow. Such a tool could lower the possibility of human error, whilst reducing the burden on hospitals relating to training, completing manual X-ray templating, and driving software packages. Necessitating no more imaging requirements over conventional manual X-ray based methods would help avoid additional costs, radiation exposure, and resource/time. Lastly, an automatic, X-ray based tool could also be of benefit in less developed countries where implant variety and access to 3D imaging would likely be more limited. The workflow of the developed size prediction tool, from inputted X-rays to size selection, is outlined. Its performance for the dimensions of five manufacturer models is then analyzed via various fit metrics and compared to the manual and semi-automatic solutions detailed above.

## 2 Materials and methods

### 2.1 Datasets

Two datasets were utilized to train and test the size prediction tool. Anterior-posterior (AP) and lateral X-ray images, as well as high resolution (3T) magnetic resonance imaging (MRI) scans, of knee joints were obtained from the Osteoarthritis Initiative (OAI) (Nevitt et al., 2006). This facilitated data to train image segmentation models within the tool and provided the necessary pairing of X-rays with 3D ground truth data to test the accuracy of the full workflow. 3D Slicer was used to segment the OAI MRI data and generate 3D bone models (Fedorov et al., 2012). Scaling of the X-ray data retrieved from the OAI dataset was completed to address geometric magnification effects and ensure consistency with the MRI data in lieu of calibration artifacts being included. CT scans with pre-segmented 3D femur and tibia models were sourced from the Korea Institute of Science and Technology Information (KISTI) (Lee and Lee, 2010). This data was used for training aspects of the size selection tool as detailed in Section 2.3. Both datasets consisted of male and female subjects with a broad range of ages. The KISTI dataset consisted purely of Asian Korean subjects, while the OAI contained White, Black and Asian Americans. No subjects used in training or testing had above mild levels of arthritis (above grade 2 out of 4) according to Kellgren and Lawrence (1956).

### 2.2 Test subjects

**FIGURE 1:**
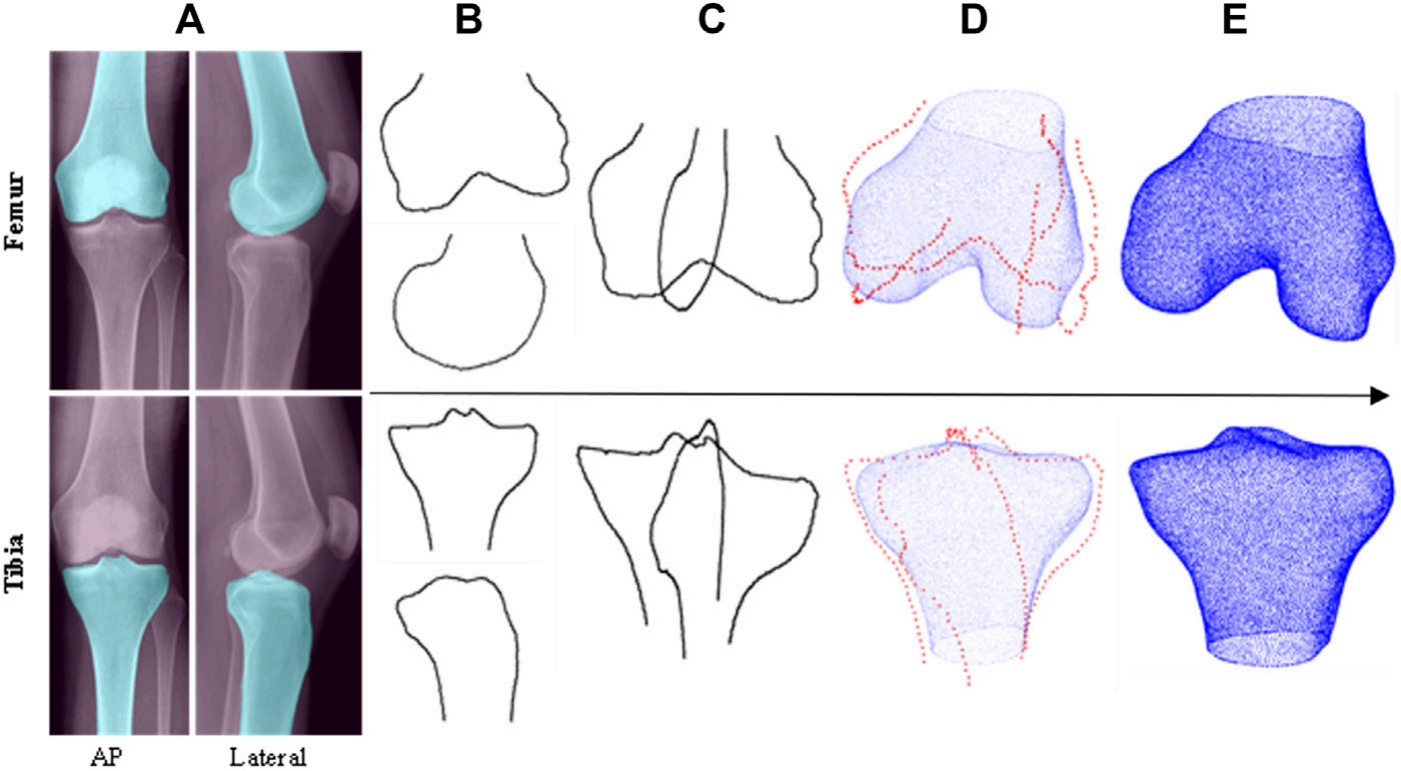
Top level workflow of the automatic 2D—3D reconstruction pipeline (top row–femur, bottom row–tibia): **(A)** CNN segmentation of input X-rays, **(B)** extracted bone contours, **(C)** contours aligned in 3D space, **(D)** PDMs used to transform contours into sparse point clouds which are then fitted to base shapes of SSMs, **(E)** 3D model predictions.

Due to the retrospective nature of the study, it was not possible to control the X-ray imaging process. Subjects’ X-rays were instead visually screened to ensure appropriate anatomical alignment and clarity in both AP and lateral projections. Figure 1 illustrates correctly captured X-rays with the scan taken straight on with both the medial and lateral condyles pointing forward in the AP projection, and side on with the posterior surfaces of the femur condyles aligned in the lateral view. 78 subjects were found to be suitable and were taken from the OAI dataset to test the size prediction tool. The test subjects ranged from 46 to 79 years old, and their demographics are summarized in Table 1. KISTI subjects were used purely for training the tool as the dataset only contained CT data without accompanying X-ray images.

**TABLE 1:** Test subject demographical information.

	Total, (%)
Overall	78
Sex	
Female	45 (57.7)
Male	33 (42.3)
Ethnicity	
White	73 (93.6)
Black	4 (5.1)
Asian	1 (1.3)
Knee	
Left	40 (51.3)
Right	38 (48.7)

### 2.3 2D—3D pipeline

Like in Zheng et al. (2018) and Massé and Ghate (2021), the size prediction tool initially utilizes inputted AP and lateral X-ray images to generate 3D estimations of patients’ femur and tibia bones before component size predictions can be made. To achieve this automatically, the 2D—3D pipeline developed in Burge et al. (2022a), Burge et al. (2022b) was utilized. The key aspects of the workflow, built using Python 3, are summarized below and illustrated in Figure 1.

**FIGURE 2:**
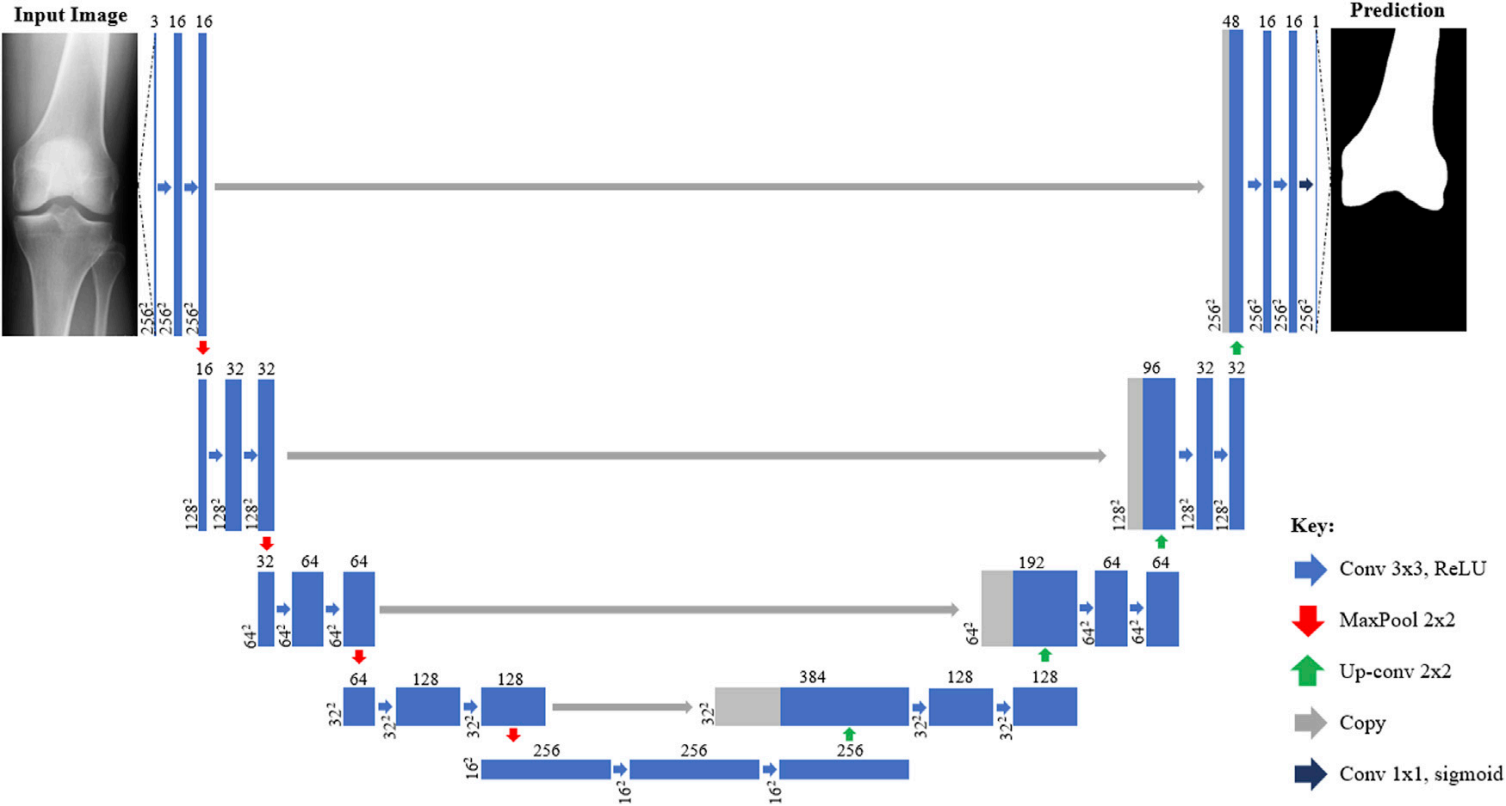
Architecture of U-Net CNN X-ray segmentation models from inputted X-ray image (left) to predicted mask (right). Each box corresponds to a multi-channel feature map with those shaded grey indicating where channels have been copied from previous layers. The number of channels is denoted on top of each box with the size provided at the lower left (256^2^ = 256 × 256 pixels). Arrows denote different operations such as convolutions.

The first step of the 2D—3D process utilizes CNNs to isolate the femur and tibia bones from the surrounding soft tissue in the inputted X-ray images, as shown in Figure 1A. Four U-Net CNN image segmentation models (one for each X-ray projection of each bone) are used in the tool. The models were built using TensorFlow and a U-Net architecture, based on Ronneberger et al. (2015), was adopted (illustrated in Figure 2). To better capture the bone profiles occupying a large proportion of the X-ray images, an additional filter resolution level (16 x 16 pixels) was incorporated. The number of filter channels used in each layer and learning rate were adjusted to achieve the best validation results. Batch size was set at 10 with 100 epochs and model weights were saved after each iteration. Once training had finished, the weights with the lowest validation loss were selected for each model to minimize overfitting. 176 X-ray image/mask pairs were developed from the OAI dataset to train each model. 20 of these (∼10%) were reserved for validation (separate to those used to test the full tool). It is noted that to build well generalized CNN models, capable of reliably segmenting any inputted X-ray, thousands of varied training images would likely be required (Liu et al., 2018). Due to the proof-of-concept nature of this study, and lack of suitable training data, it was deemed acceptable to mitigate this by adjusting the contrast/brightness settings of the inputted subject X-ray images to improve compatibility with the CNN models when testing the size prediction tool.

After segmentation, a Cany edge detector is applied to extract the four bone contours (Figure 1B) which are then aligned in 3D space as shown in Figure 1C. Point depth models (PDMs) are applied to estimate the third dimension of the points along each contour and create a sparse 3D point cloud of reference coordinates (Figure 1D). The femur and tibia reference point clouds are independently fitted to two statistical shape models (SSMs) (one for each bone) via an iterative point method. The SSMs then morph base femur and tibia shape models to fit the reference points as closely as possible. For the PDMs and SSMs, 3D bone models (20 and 100 respectively) were used from the KISTI dataset for training.

The outputs from the 2D—3D pipeline are predictions of subjects’ distal femur and proximal tibia anatomies in the form of 3D surface mesh models (Figure 1E). These can subsequently be used by the tool to approximate the most appropriately sized implant components.

### 2.4 Implant designs and sizes

**FIGURE 3:**
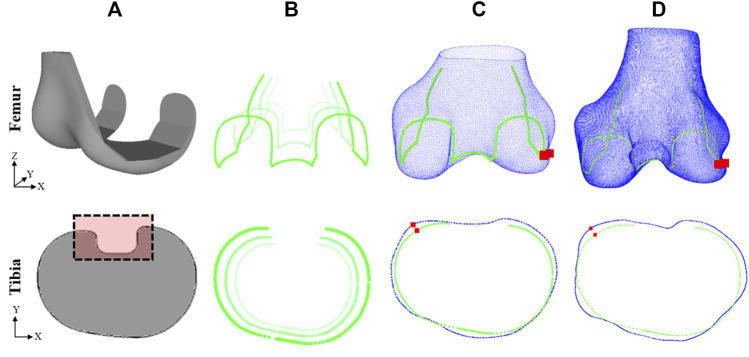
Method for evaluating fit of implant model on subject ground truth anatomy (top row–femur, bottom row–tibia): **(A)** Generic implant component designs, **(B)** edges of scaled components, **(C)** scaled components fitted to subject 3D model predictions, **(D)** scaled components fitted to subject ground truth anatomy. Dashed box in **(A)** shows detail not included in fit analysis. Red squares in **(C)** and **(D)** show the location of the maximum OUH.

Generic base models of TKR femur components and tibia plates were designed based on widely used commercial products (Figure 3A). The base models were scaled (Figure 3B) to the sizes listed for five manufacturers’ models as reported in size charts within their respective surgical technique manuals (Tables 2 and 3). This method was used as access to the official geometry of all the manufacturer models and sizes utilized within the study was not possible. Nor was acquiring physical samples for reverse engineering. Utilizing generic implant shapes also allowed for the test models to be easily controlled/edited for the purposes of the study.

**TABLE 2:** Femur component sizing chart, detailing the manufacturer, model, size identifier, and ML/AP dimensions (obtained from respective surgical technique manuals).

Zimmer Biomet (NexGen)			DePuy (Sigma)			Smith & Nephew (Legion)			Maxx Orthopedics (Freedom)			Stryker (Scorpio)		
Id.	ML (mm)	AP (mm)	Id.	ML (mm)	AP (mm)	Id.	ML (mm	AP (mm)	Id.	ML (mm)	AP (mm)	Id.	ML (mm)	AP (mm)
‘B’	58	50	‘1.5’	57	53	‘2’	58	50	‘A’	54	51	‘3’	57	51
‘C’	64	54.5	‘2’	60	56	‘3’	62	55	‘B’	58	54	‘4’	60	54
‘D’	68	58	‘2.5’	63	58	‘4’	66	59	‘C’	62	58	‘5’	62	56
‘E’	72	62	‘3’	66	61	‘5’	70	62	‘D’	64	60	‘6’	65	58
‘F’	76	66	‘4’	71	65	‘6’	73	66	‘E’	66	62	‘7’	67	61
			‘5’	73	69	‘7’	77	70	‘F’	70	66	‘8’	70	63
						‘8’	80	75	‘G’	74	70	‘9’	72	65
									‘H’	78	74	‘11’	77	70
												‘13’	82	75

**TABLE 3:** Tibia plate sizing chart, detailing the manufacturer, model, size identifier, and ML/AP dimensions (obtained from respective surgical technique manuals).

Zimmer Biomet (NexGen)			DePuy (Sigma)			Smith & Nephew (Legion)			Maxx Orthopedics (Freedom)			Stryker (Scorpio)		
Id.	ML (mm	AP (mm)	Id.	ML (mm)	AP (mm)	Id.	ML (mm)	AP (mm)	Id.	ML (mm)	AP (mm)	Id.	ML (mm	AP (mm)
‘1’	56	41	‘1’	59.2	39	‘1’	60	42	‘1’	59	40	‘3’	61	40
‘2’	62	41	‘1.5’	61.8	40.7	‘2’	64	45	‘2’	62	40	‘4’	63	42
‘3’	67	46	‘2’	64.6	42.6	‘3’	68	48	‘3’	66	42	‘5’	66	44
‘4’	70	46	‘2.5’	67.1	44.2	‘4’	71	50	‘4’	66	46	‘6’	68	45
‘5’	74	50	‘3’	69.6	45.8	‘5’	74	52	‘5’	71	48	‘7’	71	47
‘6’	77	50	‘4’	74.9	49.3	‘6’	77	54	‘6’	72	50	‘9’	77	51
			‘5’	80.6	53.1	‘7’	81	56	‘7’	76	52	‘11’	82	54
			‘6’	86.8	57.2	‘8’	85	59	‘8’	78	54	‘13’	88	58

The height of the 3D femur components was kept proportional by scaling the Z dimension in line with the AP and ML dimensions. The design of the base femur component was subtly adjusted for each model to reflect differences in transepicondylar (ML) width between the designs and ensure consistency with the dimensioning used in each of the surgical manuals. The design of the base tibia plate was kept constant and only the 2D profile was used like in prior studies (Shao et al., 2020; Clary et al., 2014). A 2D analysis was sufficient for the tibia plate because the components only interface with the resected bone on a singular 2D face.

### 2.5 Size prediction and accuracy calculation

To predict which femur component and tibia plate sizes are the most suited for a subject, the tool systematically fits each implant model and size to the 3D estimates of the subject’s anatomy using an iterative closest point method (Figure 3C). Two fit metrics were calculated after each size fitting including the global root-mean-squared error (RMSE), and local maximum OUH. The RMSE calculation was performed between the surface of the positioned component and the subject anatomy as described by Eq. 1:

$$RMS\;error=\sqrt{\frac{\sum_{i=1}^{N}{\left(x_{i}-{\hat{x}}_{i}\right)}^{2}}{N}}$$ (1)

where N is the number of points and $x_{i}-{\hat{x}}_{i}$ is the Euclidian distance between each point of the component surface and the bone. The maximum OUH was reported as the Hausdorff distance (h) anywhere between the edges of the component (C) to the edges of the bone (B), as described by Eq. 2 and shown in Figure 3. The distance (d) between each point along the component edges (c) and the bone (b) was calculated as the Euclidian distance.

$$h\left(C,B\right)=\max{\;}_{c\in C}\;\left\{\min_{\;b\in B}\;\left\{\;d\left(c,b\right)\right\}\right\}$$ (2)

For the femur, the fit analysis was completed in 3D assuming that the bone would be resected from the lateral view to the same standard dimensions as the mating faces on the femur component being fitted. For the tibia, the RMSE and maximum OUH were calculated using the 2D profile of the tibia plate and a cross-section profile taken at the intended resection point on the tibia bone. In practice, the necessary resection depth is determined by the surgeon, however, for the purposes of this study, the resection plane was located 2 mm below the height of the widest point of the tibia medial condyle, parallel with the surface of the medial plateau. Rotation of the tibia plate was limited to about its central axis and no flexion or extension was permitted. This approach consistently facilitated the largest possible surface area for stability, minimized bone loss (Schnurr et al., 2011), and enabled a continuous, flat resection plane to be created through the bone. Once RMSE and maximum OUH were recorded for each component size, the options for each model that achieved the lowest errors were outputted as the tool predictions.

### 2.6 Results analysis

After predictions were made, all sizes for each implant model were positioned on 3D ground truth models via an iterative closest point method and the resultant RMSE and maximum OUH values were calculated (Figure 3D). Ground truth models were created for each test subject by manually segmenting their MRI data as described previously. The “ground truth best” size for the individual (in terms of each fit metric, implant component, and model) was recorded as the option which resulted in the lowest calculated error when fitted to the subject’s 3D ground truth model. If the tool predicted the same as the ground truth best, the prediction was deemed to be correct. Spearman’s correlation coefficients were calculated and used to evaluate the impact on performance due to continuous variables such as subject age.

## 3 Results

### 3.1 2D—3D model generation

The accuracy of the 2D—3D process was evaluated by Burge et al. (2022b) by comparing the tool’s bone surface estimations with subjects’ 3D ground truth models. A mean RMSE of 1.09 mm (SD 0.18 mm) for the femur and 0.98 mm (SD 0.15 mm) for the tibia across the 78 subjects were reported.

### 3.2 Size selection accuracy

The accuracy of the femur component size selection for each model is reported in Table 4 and in Table 5 for tibia plates. Tables 4 and 5 demonstrate that high levels of accuracy for the size selection tool across the five implant model sizes for both implant components were obtained in terms of RMSE and maximum OUH. Minimal difference in prediction accuracy was observed across the five tibia plate models for males and females. For femur components however, males achieved 87.27% for both RMSE and maximum OUH, whilst accuracies of 71.11% and 60.44% were recorded for females respectively. Calculating Spearman’s correlation coefficients showed no strong correlations between prediction accuracies and subject age for both femur components (RMSE = −0.03, maximum OUH = 0.02) and tibia plates (RMSE = 0.21, maximum OUH = 0.25) were present.

**TABLE 4:** Femur component size prediction accuracies for each manufacturer model sizings. Size prediction accuracy is shown in terms of RMSE and maximum OUH, as well as allowing for ± one model size.

Model (no. Sizes)	RMSE correct (%)	RMSE ±1 correct (%)	Max OUH correct (%)	Max OUH ±1 correct (%)
Zimmer Biomet (5)	85.90	100.00	75.64	100.00
DePuy (6)	84.62	100.00	83.33	100.00
Smith & Nephew (7)	75.64	100.00	64.10	100.00
Maxx Orthopedics (8)	73.08	100.00	65.38	100.00
Stryker (9)	70.51	98.72	70.51	97.44
Mean	77.95	99.74	71.79	99.49

**TABLE 5:** Tibia plate size prediction accuracies for each manufacturer model sizings. Size prediction accuracy is shown in terms of RMSE and maximum OUH, as well as allowing for ± one model size.

Model (no. Sizes)	RMSE correct (%)	RMSE ±1 correct (%)	Max OUH correct (%)	Max OUH ±1 correct (%)
Zimmer Biomet (6)	87.18	100.00	74.36	98.72
DePuy (8)	85.90	100.00	82.05	100.00
Smith & Nephew (8)	65.38	100.00	62.82	98.72
Maxx Orthopedics (8)	83.33	98.72	69.23	97.44
Stryker (8)	80.77	100.00	75.64	100.00
Mean	80.51	99.74	72.82	98.98

### 3.3 Implant fit

For the sizes deemed to be the best by the size prediction tool for each model and each of the test subjects, the resulting fit on the ground truth models were recorded for both metrics. These results are shown in Table 6 for femur components and Table 7 for tibia plates, alongside the best possible results. The tables show that the results obtained using the predictions made by the tool for both component types were on average only marginally inferior to the best possible outcomes in terms of both RMSE and maximum OUH.

**TABLE 6:** Femur component mean RMSE and mean maximum OUH results for ground truth (GT) best sizes and predictions, split by manufacturer model sizings.

Model (no. Sizes)	Mean GT best RMSE (mm)	Mean prediction RMSE (mm)	Mean GT best max OUH (mm)	Mean prediction max OUH (mm)
Zimmer Biomet (5)	1.26	1.31	3.55	3.70
DePuy (6)	1.06	1.09	2.95	3.02
Smith & Nephew (7)	1.05	1.14	2.89	3.29
Maxx Orthopedics (8)	0.99	1.04	2.67	2.91
Stryker (9)	1.03	1.10	2.87	3.16
Mean	1.08	1.13	2.99	3.22

**TABLE 7:** Tibia plate mean RMSE and mean maximum OUH results for ground truth (GT) best sizes and predictions, split by manufacturer model sizings.

Model (no. Sizes)	Mean GT best RMSE (mm)	Mean prediction RMSE (mm)	Mean GT best max OUH (mm)	Mean prediction max OUH (mm)
Zimmer Biomet (6)	1.73	1.77	3.48	3.63
DePuy (8)	1.11	1.19	2.52	2.70
Smith & Nephew (8)	1.11	1.25	2.53	2.85
Maxx Orthopedics (8)	1.31	1.38	2.91	3.20
Stryker (8)	1.10	1.21	2.48	2.71
Mean	1.27	1.36	2.78	3.02

**FIGURE 4:**
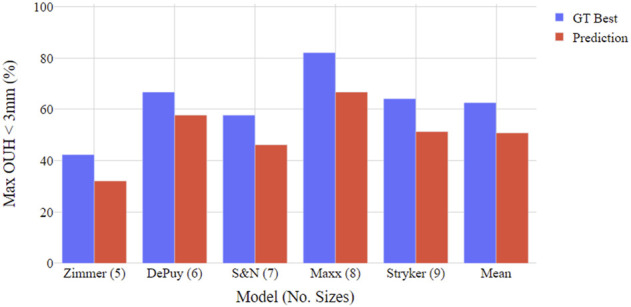
Boxplot illustrating proportion of test subjects achieving maximum OUH < 3 mm for various femur component model sizings. Both ground truth (GT) best possible size and tool prediction results shown.

**FIGURE 5:**
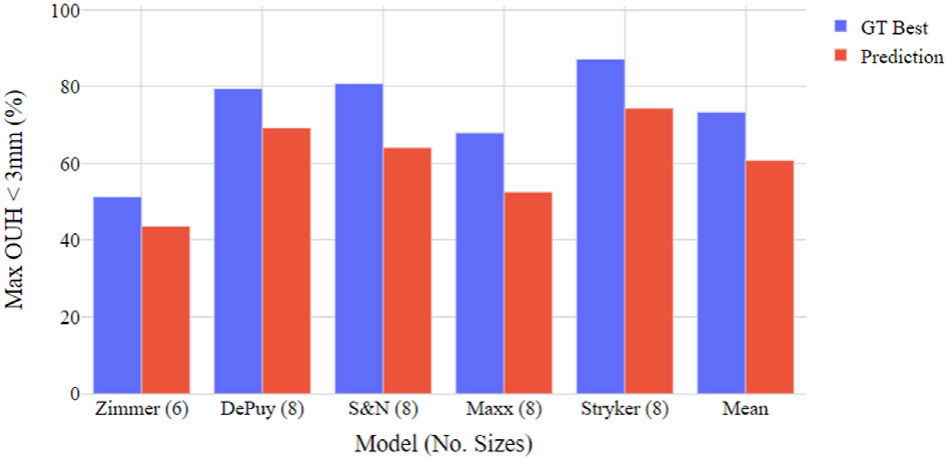
Boxplot illustrating proportion of test subjects achieving maximum OUH < 3 mm for various tibia plate model sizings. Both ground truth (GT) best possible size and tool prediction results shown.

Figures 4, 5 show the proportion of subjects achieving maximum OUH <3 mm for the various femur component and tibia plate model sizes. The figures demonstrate that the level of subjects attaining below the threshold was on average 10% less for femur components compared to tibia plates and the levels varied significantly between the different manufacturer model sizes. The proportion of subjects seeing maximum OUH of ≥3 mm was on average 12% higher than the ground truth best when the tool’s size predictions were used.

## 4 Discussion

The most important outcome from this study is that a fully automatic, X-ray based TKR size prediction tool was developed and shown to be reliable for a range of implant model sizes and test subjects. For both femur components and tibia plates, the tool more accurately predicted the ground truth best size in terms of RMSE compared to maximum OUH. Nevertheless, the accuracies for both metrics were consistently high with the ground truth best size predicted on average 78% of the time (in terms of RMSE) and 72% of the time (in terms of maximum OUH) for femur components. For tibia plates the prediction accuracies were 81% in terms of RMSE and 73% in terms of maximum OUH. These increased to 99–100% for ±1 size for both metrics and component types. The prediction accuracy of the tool was not found to be sensitive to subject age, nor was it sensitive to sex for predicting tibia plate size. However, 16–27% higher accuracies were achieved for male subject femur component size selection. This was likely due to the large White American male dimensions in the test population– often requiring the upper limits of the femur component size ranges or beyond. Females on the other hand utilized a broader range of smaller sizes which made predicting the correct option more challenging. Using a more balanced group of subjects, featuring ethnicities typically requiring smaller implant sizes such as Asian Chinese (Li et al., 2019), would likely reduce this effect and result in similar prediction accuracies between sexes.

Another key finding was the proportion of subjects obtaining clinically significant levels of OUH, even when the best possible sizes were used, was above 30% for all implant models evaluated. This aligns with results reported by previous studies (Wernecke et al., 2012; Mahoney and Kinsey, 2010) where the performance of non-customised implants, particularly with a limited number of size options, was shown to be poor. In this study, the model with the fewest number of sizes (the Zimmer Biomet NexGen), performed the worst in terms of the resulting mean RMSE, maximum OUH, and proportion of subjects achieving maximum OUH <3mm, whilst models with more sizes generally performed better. Nevertheless, for the tool’s size prediction accuracies, the opposite was found. The Zimmer Biomet NexGen (5/6 sizes) obtained the best results, whilst the Stryker Scorpio (9 sizes) achieved the lowest accuracy for femur components (both in terms of RMSE). Comparing the results for the Smith & Nephew Legion and the Maxx Orthopedics Freedom tibia plate sizes showed considerable differences in prediction accuracies and resulting fits, despite an equal number of sizes. It is again emphasized that these results do not reflect the true performance of the named manufacturer models as generic base models were used in the analysis. However, the importance of selecting the optimum sizing dimensions, not just enough options, is highlighted. For models with many similar sizes, it was often observed that when the tool failed to predict the best possible size, the optimal component dimensions would have been close to the center of two options. As a result, the resulting RMSE and/or maximum OUH for the predicted size were only marginally worse. To better align with the approach taken by surgeons in said scenarios, the tool could be configured to always downsize the component when minimal difference in fit is predicted between two sizes (Dai et al., 2014).

Comparing the results to those available in the literature for manual planning/templating, the size prediction accuracy of the tool was shown to be considerably better than the average reported across the 10 studies summarized by Hernández-Vaquero et al. (2019). This was also true when compared to studies that utilized CT data for manual templating (Vaishya et al., 2018; Kobayashi et al., 2012). Therefore, due to the high accuracies obtained without the need for user feedback or training, it is anticipated that hospitals could realize considerable time and resource savings and reduce the possibility of human error by adopting the tool. In terms of other computational approaches, comparing the tool to results detailed previously for prediction models based on subject demographics (Trainor et al., 2018; Sershon et al., 2017) showed significantly improved performance was possible by using the approach outlined in this study. The tool achieved a better average tibia plate size prediction accuracy and matched the level reported for femur components (in terms of RMSE) when compared to the solution published by Zheng et al. (2018). When compared to the results reported by Massé and Ghate (2021), the tool was on average >20% more accurate in selecting the best size for both component types. This study has therefore shown that fully automating the size prediction tool did not compromise accuracy, but similar or better results were in fact obtained. Moreover, the tools developed by Zheng et al. (2018) and Massé and Ghate (2021) were only tested on 23 and 45 subjects respectively, compared to 78 in this work. The authors also only used single implant models and one global fit metric in the cited studies. By exploring a range of implant model sizes and employing both global and clinical fit metrics to evaluate performance, this study has provided a more comprehensive assessment of the developed tool’s robustness and applicable use with various products.

The performance of the tool could be further improved by controlling the alignment and quality of the inputted X-ray images to ensure compatibility with the 2D—3D pipeline (Burge et al., 2022b). Due to the datasets available, the testing of the tool was limited to predominantly White Americans without severe arthritis degeneration, whilst the modules of the tool were principally trained using Asian Korean data. Differences in performance between sexes and with age were evaluated in the study, however, with the data available it was not possible to analyze potential biases across ethnicities and/or the impact of arthritis severity on performance. For the development of future implant size prediction tools, it is encouraged that larger datasets, featuring hundreds of subjects with various ethnicities, ages, sexes and Kellgren and Lawrence grades, all imaged with consistent alignments/scan settings, be obtained. This would help better generalize the CNN models and improve robustness across the full range of potential patient demographics. Moreover, to further reduce overfitting, regularization techniques such as drop-out layers could be incorporated into the CNN model architecture. It should be noted that the method for determining the best fitting implant designs varied between the studies referenced. The best size in most of the referenced studies was determined by a medical professional during or after surgery. This study however was completed without clinical work and used computational fit metrics to determine performance (like in Zheng et al. (2018)). Future work could seek to test the size prediction tool against size choices made during real surgical procedures to provide a more realistic means for comparison. Furthermore, generic implant designs, scaled to published manufacturer model size charts, were used instead of official geometries. Going forwards, it is hoped manufacturers will adapt the framework of the tool for use with commercially available products.

## 5 Conclusion

In this study a computational TKR size prediction tool was developed that uses two X-ray images to assist clinicians with selecting the best implant sizes for patients. The tool achieved selection accuracies superior to those reported for manual templating, as well as when compared to other computational alternatives. By removing the need for manual templating or guiding semi-automatic software tools, the tool could minimize the time and resource required for TKR preoperative assessment, increase surgeons’ confidence in the outcomes, and minimize the possibility of size changes during surgery, whilst still achieving reliable size prediction results. This could also help reduce surgery time, minimize the level of component inventory required, lessen surgeon accountability for determining the right size implants, and ultimately improve outcomes for patients. Finally, the tool could be used to assess the need for customised solutions, or for a bespoke selection of implants depending on patient morphology.

## Data Availability

The datasets presented in this article are not readily available because access to the data used in the study is restricted and controlled by the OAI and KISTI. Requests to access the datasets should be directed to https://nda.nih.gov/oai/and https://www.kisti.re.kr/eng/.
